# Does food responsiveness change in people with first episode psychosis (FEP) over a period of 6 months after commencing antipsychotics? Preliminary results

**DOI:** 10.1192/bjo.2021.682

**Published:** 2021-06-18

**Authors:** Adrian Heald, Mark Shakespeare, Kevin Williamson, Adrianne Close, Adrian Phillipson, Suzanne Higgs

**Affiliations:** 1 Salford Royal Foundation Trust; 2 RDASH; 3 University of Birmingham

## Abstract

**Aims:**

We here present preliminary results from our study to understand better the changes in people’ s experience of food in the months after diagnosis with first episode psychosis (FEP). Weight gain often occurs in the weeks/months after diagnosis and is related to an increase in appetite and food intake. Many drugs that are effective in treating psychosis are associated with changes in the way that people experience reward when they eat.

The aim of this project is to increase our understanding of exactly why this happens in terms of an individual's experience of food reward and reduced satiety – and therefore how we can help people with FEP to keep their weight down. At this stage we are looking at the feasibility of applying currently available evaluation tools to people in this situation.

**Method:**

A convenience sample was used to recruit 10 service users from RDaSH NHS FT Early Intervention Services. This is a feasibility study which will provide data to underpin a fully powered, larger trial.

Rating scales applied were:

Power of food questionnaire: measures responsiveness to the food environment.

Intuitive Eating Scale: measures an individual's tendency to follow their physical hunger and satiety cues.

The loss of control over eating scale (LOCES): measures a global sense of whether individuals experience LOC over eating.

Dutch Eating Behaviour Questionnaire (DEBQ): measures restrained eating, emotional eating and external eating.

**Result:**

The ages of the participants ranged from 17-26 years. All were started on Olanzapine at the dose of 5 or 10 mg daily.

Baseline total scores for the Power of Food (2.47-3.80)/5 (higher score = more responsiveness) and Intuitive Eating scales (2.10-2.62)/5 (higher score = greater tendency to follow hunger and satiety cues) were in the mid-range, while the LOCES scores varied widely from 1.50-2.38/5.

The DEBQ restrained subscale score range was 2.40-2.80/5 (higher indicates greater restraint with food) while the DEBQ external subscale ranged from 2.70—3.00/5 (higher = greater tendency to overeat) and the DEBQ emotional subtotal score was 1.92-1.94/5, in keeping with a relatively low emotional drive to eat.

**Conclusion:**

Our preliminary results reveal at the beginning of antipsychotic treatment a moderate responsiveness to food and tendency to follow hunger/ satiety cues, with scores for Loss of Control of eating in the low to moderate range and a low emotional drive to eat. The difference between these and the follow-up eating behaviour scores will provide important clues as to the precise changes in eating behaviour with anti-psychotic treatment in FEP.

